# Transcriptomic profiling of *Melon necrotic spot virus*-infected melon plants revealed virus strain and plant cultivar-specific alterations

**DOI:** 10.1186/s12864-016-2772-5

**Published:** 2016-06-07

**Authors:** Cristina Gómez-Aix, Laura Pascual, Joaquín Cañizares, María Amelia Sánchez-Pina, Miguel A. Aranda

**Affiliations:** Departamento de Biología del Estrés y Patología Vegetal, Centro de Edafología y Biología Aplicada del Segura (CEBAS) – CSIC, apdo. correos 164, 30100 Espinardo, Murcia Spain; Instituto de Conservación y Mejora de la Agrodiversidad Valenciana (COMAV) – UPV, Camino de Vera s/n, 46022 Valencia, Spain; Centre for Research in Agricultural Genomics CRAG, CSIC-IRTA-UAB-UB, Campus 10 UAB Bellaterra, 08193 Barcelona, Spain

**Keywords:** MNSV, CMV, Cucurbits, *Cytokinin-O-glucosyltransferase*, Resistance, WMV

## Abstract

**Background:**

Viruses are among the most destructive and difficult to control plant pathogens. Melon (*Cucumis melo* L.) has become the model species for the agriculturally important *Cucurbitaceae* family. Approaches that take advantage of recently developed genomic tools in melon have been extremely useful for understanding viral pathogenesis and can contribute to the identification of target genes for breeding new resistant cultivars. In this work, we have used a recently described melon microarray for transcriptome profiling of two melon cultivars infected with two strains of *Melon necrotic spot virus* (MNSV) that only differ on their 3′-untranslated regions.

**Results:**

Melon plant tissues from the cultivars Tendral or Planters Jumbo were locally infected with either MNSV-Mα5 or MNSV-Mα5/3’264 and analysed in a time-course experiment. Principal component and hierarchical clustering analyses identified treatment (healthy *vs.* infected) and sampling date (3 *vs.* 5 dpi) as the primary and secondary variables, respectively. Out of 7566 and 7074 genes deregulated by MNSV-Mα5 and MNSV-Mα5/3’264, 1851 and 1356, respectively, were strain-specific. Likewise, MNSV-Mα5/3’264 specifically deregulated 2925 and 1618 genes in Tendral and Planters Jumbo, respectively. The GO categories that were significantly affected were clearly different for the different virus/host combinations. Grouping genes according to their patterns of expression allowed for the identification of two groups that were specifically deregulated by MNSV-Mα5/3’264 with respect to MNSV-Mα5 in Tendral, and one group that was antagonistically regulated in Planters Jumbo vs. Tendral after MNSV-Mα5/3’264 infection. Genes in these three groups belonged to diverse functional classes, and no obvious regulatory commonalities were identified. When data on MNSV-Mα5/Tendral infections were compared to equivalent data on cucumber mosaic virus or watermelon mosaic virus infections, *cytokinin-O-glucosyltransferase2* was identified as the only gene that was deregulated by all three viruses, with infection dynamics correlating with the amplitude of transcriptome remodeling.

**Conclusions:**

Strain-specific changes, as well as cultivar-specific changes, were identified by profiling the transcriptomes of plants from two melon cultivars infected with two MNSV strains. No obvious regulatory features shared among deregulated genes have been identified, pointing toward regulation through differential functional pathways.

**Electronic supplementary material:**

The online version of this article (doi:10.1186/s12864-016-2772-5) contains supplementary material, which is available to authorized users.

## Background

Viruses change the physiology and metabolism of infected plants, and can directly or indirectly influence the host’s gene expression patterns [1–4]. High-throughput technologies such as expressed sequence tags (ESTs), microarrays and next-generation sequencing, have made possible the simultaneous analysis of functional data for many genes and the study of the plant’s transcriptomic remodelling in response to virus infections [5]. In the past few years, DNA microarrays have become popular tools for comparative high-throughput gene expression analysis, and microarray platforms have become available for both model and non-model crop species. Melon (*Cucumis melo* L.), in addition to its agronomic importance, has biological features that make it an interesting experimental model, favouring the development of a growing number of genetic and molecular tools for this species, including large ESTs collections [6, 7], TILLING platforms [8, 9] and the sequencing of its genome [10]. More specifically, EST sequencing has allowed the development of a melon-specific microarray [11], which has been used for transcriptomic profiling of *Cucumber mosaic virus* (CMV), *Watermelon mosaic virus* (WMV) and *Monosporacus cannonballus*-infected plants [11–13]. In this work, we have used the melon microarray to profile the melon transcriptome after infection with *Melon necrotic spot virus* (MNSV).

MNSV (genus *Carmovirus*, family *Tombusviridae*) is endemic in cucurbit crops worldwide, often causing significant economic losses due to epidemic outbreaks. The MNSV genome is composed of a 4.3Kb, single-stranded positive-sense RNA containing at least five open reading frames (ORFs) [14] which are flanked by two untranslated regions (UTRs) at their 5′ and 3′ termini. The 3′ ORF encodes the capsid protein (CP) which has a structural role, is necessary for vascular transport of the virus, plays a role in suppression of RNA silencing [15] and is involved in virus transmission [16]. It also contains a double gene block (DGB), typical of carmoviruses, consisting of two small, centrally located ORFs, which encode two consecutive 7 kDa proteins (p7A and p7B) involved in the cell-to-cell movement of the virus [15, 17]. The 5′ ORF can either encode a 29 kDa protein (p29) ending in an amber codon, or a larger 89 kDa gene product (p89) if it is read-through, which contains the RNA-dependent RNA polymerase (RdRp) domain. The p29 and p89 proteins are involved in viral replication [14, 15], which takes place in virus-altered mitochondria [18]. The 3′-UTR of genomic MNSV RNAs, which are identical to those of sub-genomic RNAs, has been shown to contain sequences that act as cap-independent translational enhancers (3′-CITEs) [19, 20]. Depending on the specific nature of these 3′-CITEs, MNSV can infect *N. benthamiana* and the otherwise resistant melon plants that carry the recessive *eIF4E*^228L^ allele at the *nsv* locus [20–22]. Interestingly, 3′-CITEs exhibit a modular nature, as they can be exchanged among viral strains or even viral species through recombination [19, 23].

In this work, we have used two MNSV strains that only differed in their 3-UTRs, namely, MNSV-Mα5 and a chimera with its 3′-UTR from MNSV-264 (MNSV-Mα5/3’264) for infection profiling. MNSV-264 is a strain that is able to break the resistance controlled by *nsv* [21, 23]. The characterization of melon cultivar-specific responses was also investigated, and two melon cultivars were used for this purpose. These were: cv. Tendral, which is fully susceptible to MNSV, and cv. Planters Jumbo, which is homozygous for the recessive *eIF4E*^228L^ resistance allele and is therefore resistant to most MNSV isolates but not to those carrying the MNSV-264 3′-CITE [21], as is the case for MNSV-Mα5/3’264 [23]. Locally-infected tissues were analysed in a time-course experiment and the melon microarray [11] was used for describing differential alterations of the melon transcriptome associated with: (i) the presence of one or another 3′-UTR in the MNSV RNA, (ii) the melon genotype and (iii) the type of plant tissue infected.

## Results

### Identification of the main sources of variability

**Fig. 1:**
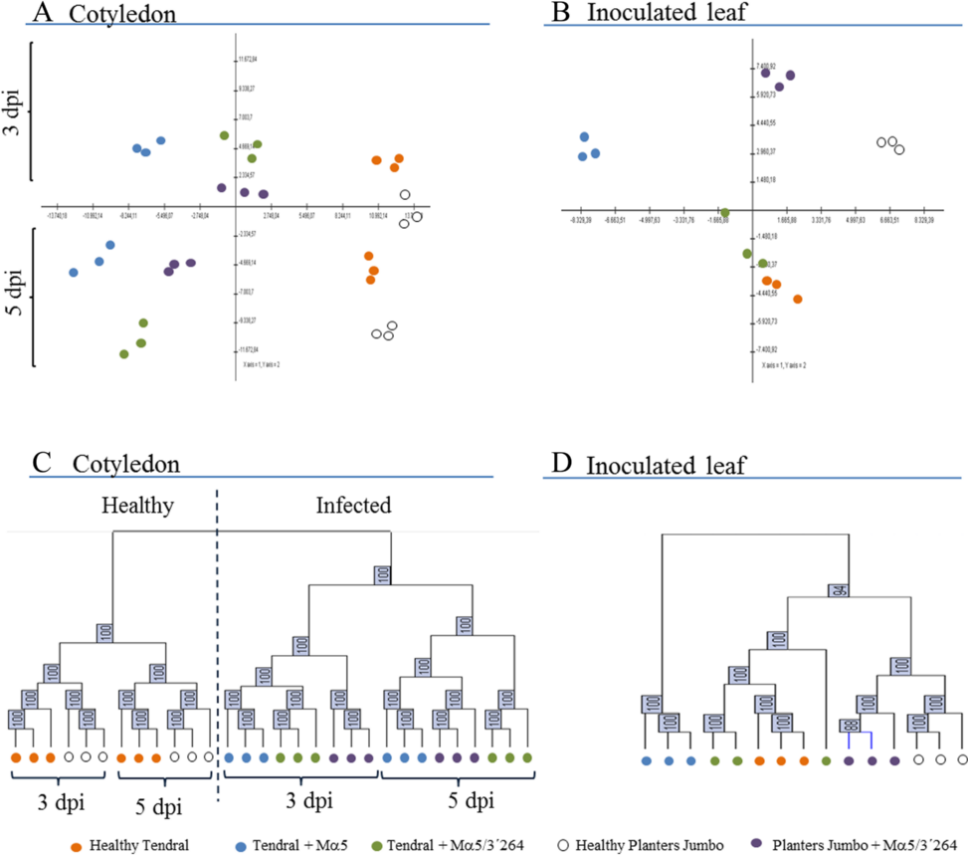
Analysis of biological variability in microarray samples. **a–b** Principal component analysis (PCA) of cotyledon samples at 3 and 5 days post inoculation (dpi) (A), and directly inoculated leaf at 5 dpi (B), for the Tendral and Planters Jumbo cultivars analyzed after normalization of microarray data. **c–d** Dendrogram obtained after clustering cotyledon and directly-inoculated leaf samples. Bootstrap values are shown in the boxes. The first two axes of the PCA accounted for 69.43 % (PC-1 = 49.27 % and PC-2 = 20.16 %) of the variability in the data in A and for 68.03 % (PC-1 = 39.82 % and PC-2 = 28.21 %) in B

Once the data from each sample were normalized, biological variability and sample grouping were analyzed using principal component analysis (PCA) (Fig. 1a and b). Biological replicates from infected tissues, either from cotyledons (Fig. 1a) or leaves (Fig. 1b), always grouped together. The first component of variance separated cotyledon samples by treatment (healthy *vs.* infected) and the second one by time after infection (3 *vs.* 5 dpi). Interestingly, Tendral cotyledon or leaf samples inoculated with MNSV-Mα5 separated from their healthy controls to a greater degree than the rest of the infected *vs.* healthy pairs (Fig. 1a and b); in contrast, Tendral leaf samples inoculated with MNSV-Mα5/3’264 separated to a lesser degree from their healthy controls than the other pairs (Fig. 1b). A hierarchical clustering analysis was also performed (Fig. 1c and d), and the results showed that once again, the cotyledon samples clustered primarily by treatment (healthy *vs.* infected) and then by time after infection (3 *vs.* 5 dpi). Among cotyledon samples, clustering varied for 3 and 5 dpi, with Tendral samples infected with the two viral isolates becoming more distinct with time (Fig. 1a and 1c). In the case of the inoculated leaves, the differentiation between infected and non-infected samples was less clear as compared to the cotyledon samples, especially for Tendral leaves inoculated with MNSV-Mα5/3’264. As for cotyledons, Tendral leaves inoculated with MNSV-Mα5 showed the greatest differentiation as compared to the controls (Fig. 1b and 1d), suggesting greater transcriptomic changes in this cultivar by MNSV-Mα5 than in the other cases.

According to these results, MNSV-Mα5 induced faster and more marked changes in Tendral as compared to MNSV-Mα5/3’264, an effect that could also be seen in inoculated leaves. Among cultivars, the course of the infection resulted in greater differentiation among the samples of the different cultivars inoculated with the same virus.

### Transcriptomic remodeling in inoculated cotyledons

#### Progression of MNSV accumulation

**Fig. 2:**
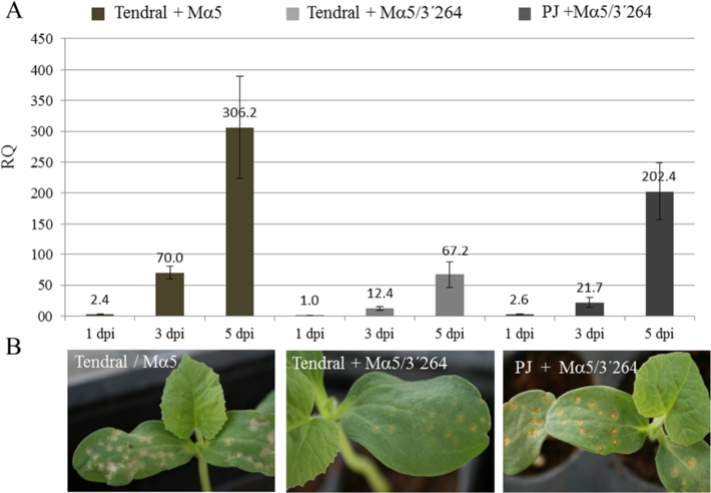
Relative quantification of viral RNA and symptoms in melon cotyledons infected with Melon necrotic spot virus (MNSV). **a** RNA accumulation as measured by quantitative polymerase chain reaction of MNSV-Mα5 (Mα5) and MNSV-Mα5/3’264 (Mα5/3’264) in melon cotyledon of the Tendral cultivar and MNSV-Mα5/3’264 in melon cotyledons of the Planters Jumbo (PJ) cultivar. Tendral samples infected with MNSV-Mα5/3’264 at 1 day post-inoculation (dpi) were used as calibrators for relative quantification. **b** MNSV induced symptoms in melon cotyledons at 7 days post inoculation

The quantification of the accumulation of each virus isolate in inoculated cotyledons was done through RT-qPCR at 1, 3 and 5 dpi (Fig. 2a). An increase in viral accumulation was observed from 1 to 5 dpi in every virus/host combination, although virus accumulation was lower in the case of Tendral inoculated with MNSV-Mα5/3’264 (Fig. 2a). The differences in accumulation were already detected from the first sampling date, where higher accumulation was observed in Tendral/MNSV-Mα5 and Planters Jumbo/MNSV-Mα5/3’264 combinations with respect to Tendral/MNSV-Mα5/3’264. For microarray analysis, we used sampling time points 3 and 5 dpi. Note that the relative increase in virus accumulation between these two time points was similar for Tendral/MNSV-Mα5 and Tendral/MNSV-Mα5/3’264 (approx. fivefold) but larger for Planters Jumbo/MNSV-Mα5/3’264 (approx. tenfold). The development of symptoms (Fig. 2b) induced by MNSV-Mα5/3’264 on Tendral cotyledons showed a slower progression as compared to the symptoms induced by MNSV-Mα5 in the same cultivar and of those induced by MNSV-Mα5/3’264 in Planters Jumbo.

#### Differentially expressed genes during infection progression

**Fig. 3:**
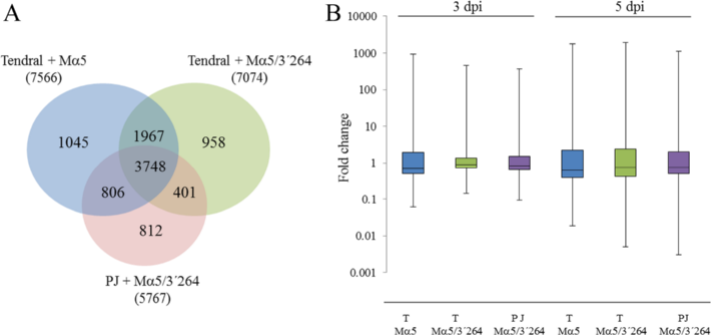
Differentially expressed genes as a function of time. **a** Venn diagrams of differentially expressed genes as a function of time identified by maSigPro in the three different virus/host combinations. In blue, differentially expressed genes in Tendral cotyledons infected with MNSV-Mα5 (T + Mα5). In green, differentially expressed genes in Tendral cotyledons infected with MNSV-Mα5/3’264 (T + Mα5/3’264). In pink, differentially expressed genes in Planters Jumbo cotyledons infected with MNSV-Mα5/3’264 (PJ + Mα5/3’264). **b** Broad gene expression trends in samples analyzed by microarray. Gene expression fold changes were calculated for deregulated unigenes identified by microarray analysis and used to construct box plots for each cultivar/virus/days post-inoculation (dpi) combination

To analyze differentially expressed genes as a function of time and virus isolate, we used the microarray Significant Profiles package (maSigPro) [24]. In Tendral, we identified 7566 differentially expressed genes that were associated to infection by MNSV-Mα5, and 7074 genes associated to infection by MNSV-Mα5/3’264, compared to 5767 deregulated by the latter in Planters Jumbo (Additional file 1), with all virus/host combinations causing common as well as specific changes (Fig. 3a). The magnitude of deregulation at 3 dpi was greater in MNSV-Mα5-infected Tendral plants, while at 5 dpi MNSV-Mα5/3’264 induced greater deregulation (Fig. 3b).

**Fig. 4:**
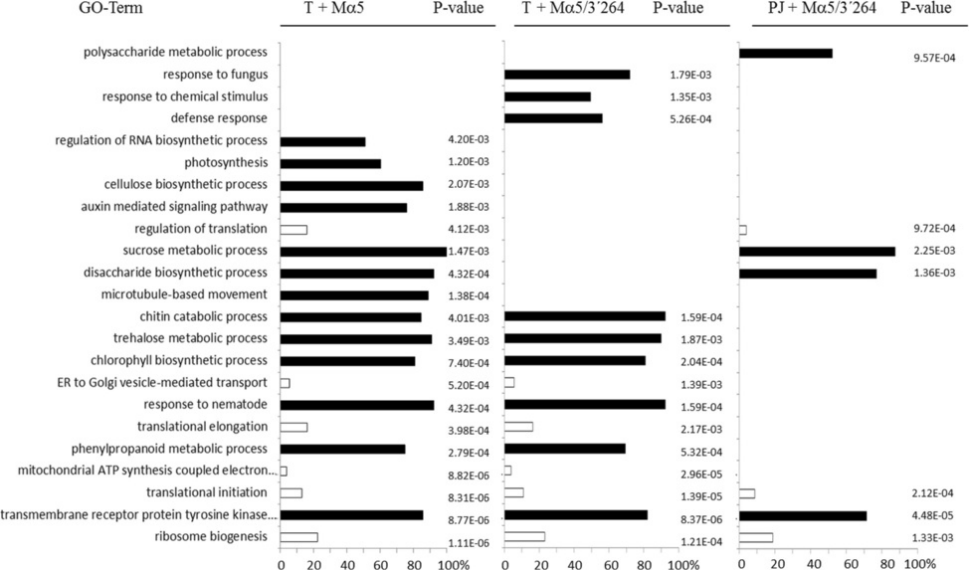
Significant Gene Ontology (GO) categories of biological processes among the deregulated unigenes identified in each virus/host combination. Differentially-expressed unigenes identified by the microarray analysis of cotyledon samples, over-represented (black) and under-represented (white). Percentage of deregulated unigenes from the total number of unigenes included in each GO category is indicated on the horizontal axis

To identify the main biological processes affected by each infection as a function of time, the Gene Ontology terms (GO terms) of the differentially expressed genes were analyzed with the Blast2GO program [25]. In agreement with the number of deregulated genes, a greater number of over- and under-represented GO categories were found during infection with MNSV-Mα5 than with MNSV-Mα5/3’264 in Tendral. Between cultivars, the number of identified terms was lower in Planters Jumbo than in Tendral (Additional file 1). Importantly, the GO categories that were significantly affected were clearly different for the different virus/host combinations, with some commonalities but many differences among host/virus treatments (Fig. 4). For instance, on the MNSV-Mα5 list, we found GO terms related to auxin signaling and microtubule-mediated movement as specifically represented. Among the terms shared with MNSV-Mα5/3’264, we found over-represented terms that were related to the photosynthesis and chloroplast (Fig. 4). Other terms were statistically significant only for Tendral infected with MNSV-Mα5/3’264; among them we found many related to response to stress, response to fungus or chemical stimulus. On the Planters Jumbo list, the most important under-represented terms were related to translation and ribosome biogenesis, which were also present on the other two lists (Fig. 4; Additional file 1). In conclusion, differential transcriptomic remodeling not only referred to the number of affected genes, but also to the nature of the biological processes involved.

#### Virus-specific transcriptomic alterations

**Fig. 5:**
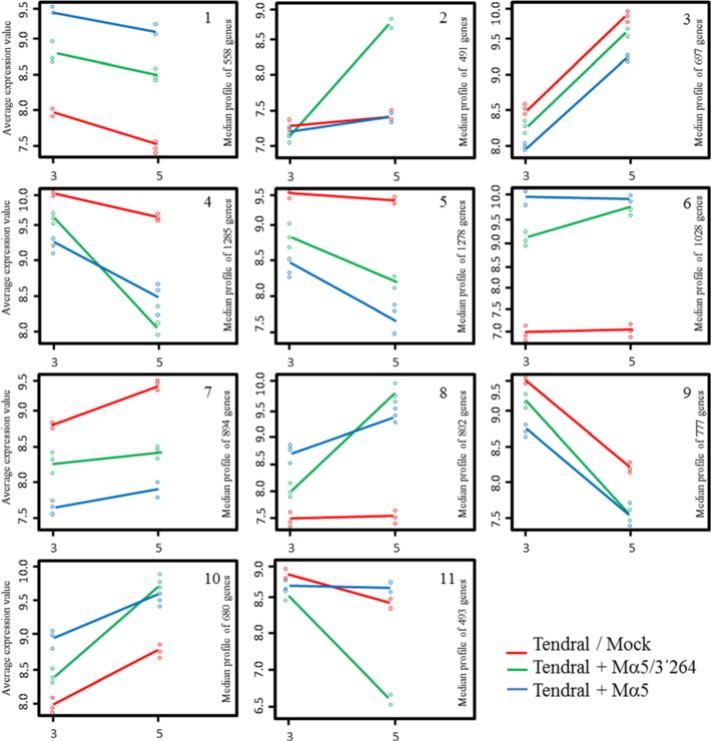
Clustering of genes that changed during the development of the MNSV infection in Tendral cotyledons. The expression patterns of the mock and infected Tendral cotyledons with MNSV-Mα5 and MNSV-Mα5/3’264 are represented by separated lines. The average expression value is represented on the y-axis. Different time points of sampling are represented on the x-axis (3 and 5 dpi)

MNSV-Mα5 and MNSV-Mα5/3’264 deregulated a great number of genes that were specific to each virus in the same cultivar, 1851 (1045 + 806) and 1359 (958 + 401), respectively (Fig. 3a). The functional analysis of these genes did not find statistically significant GO terms. Genes deregulated by both viruses in Tendral were grouped according to their expression level by using the *k*-means algorithm [26]. The gene’s patterns of expression allowed the identification of 11 groups, where most of the genes showed changes in expression at 3 dpi with respect to the uninfected control, with these changes accentuated with time (Fig. 5). Most of the groups identified included genes that were co-regulated by the two viral strains; groups 1, 6, 8 and 10 were composed of genes that were upregulated by both viruses with respect to the control, and groups 4, 5, 7 and 9 were composed by the inhibited genes (Fig. 5). A greater measurable deregulation of genes was found for MNSV-Mα5 than for MNSV-Mα5/3’264, although at 5dpi the expression levels were either equal or greater in the case of MNSV-Mα5/3’264 (Fig. 5). Among the genes repressed by MNSV-Mα5 and MNSV-Mα5/3’264, many terms related to functions associated to chloroplasts and photosynthesis were found (Additional file 2).

**Fig. 6:**
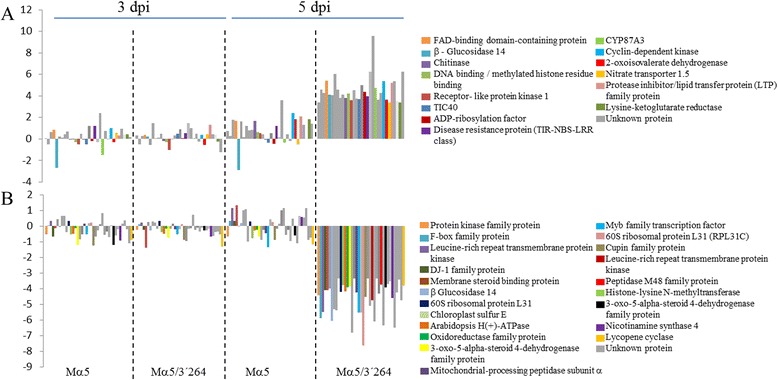
Differentially expressed genes included in clusters 2 and 11, respectively, of Tendral with fold changes above 10. **a** Unigenes included in cluster 2 that are activated in Tendral cotyledons infected with MNSV-Mα5/3’264 at 5 dpi but not by MNSV-Mα5. **b** Unigenes included in cluster 11 that are inhibited in Tendral cotyledons infected with MNSV-Mα5/3’264 at 5 dpi but not by MNSV-Mα5

Remarkably, among the 11 groups, groups 2 and 11 were composed of genes that were specifically activated or inhibited, respectively, by MNSV-Mα5/3’264 (Fig. 5). A manual analysis of the genes in group 2 confirmed the functional diversity of the genes found, with genes such as those related to response to hormones such as auxins, giberellins, indole-acetic acid, as well as transcription factors and translation elongation present (Additional file 2). Selecting the genes with a fold change above 10 reduced the list to 32 genes with largely unknown functions (Fig. 6a). Among the results, the unigene cCL2380Contig1, of unknown function, stood out, as in the samples infected with MNSV-Mα5/3’264 it was deregulated over 700 times with respect to its control (Log_2_FC-9.58; Fig. 6a; Additional file 2). Among the genes in group 11 that were inhibited by this virus, that hardly suffered modifications due to MNSV-Mα5 infection, we found numerous ribosomal proteins, Myb-family transcription factors, various oxidoreductases and proteins involved in steroid metabolism (Fig. 6b; Additional file 2).

#### Cultivar-specific transcriptomic alterations

**Fig. 7:**
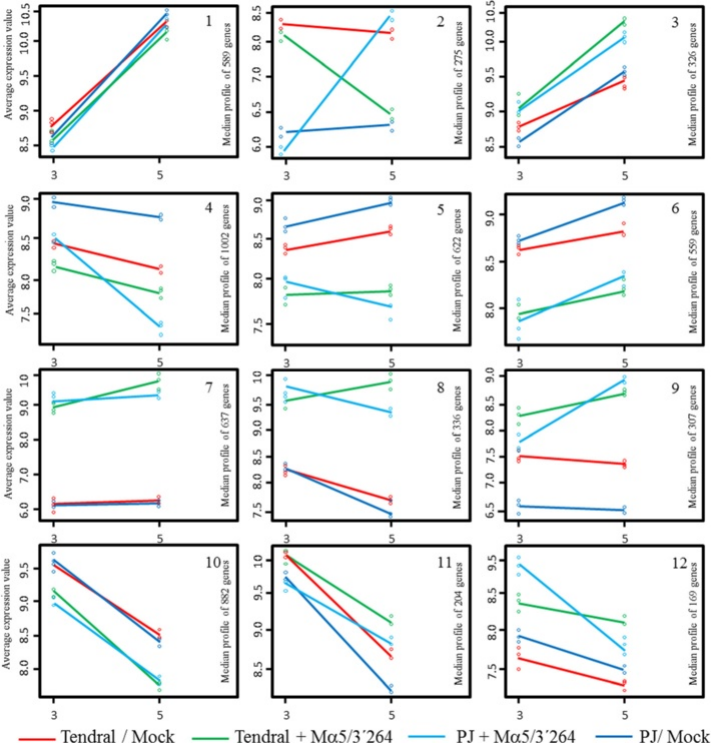
Clustering of genes that changed during the development of MNSV infection in Planters Jumbo and Tendral cotyledons. The expression patterns of the mock and infected Tendral and Planters Jumbo cotyledons with MNSV-Mα5/3’264 are represented by separated lines. The average expression value is shown on the y-axis. Different time points of sampling are shown in the x-axis (3 and 5 dpi)

**Fig. 8:**
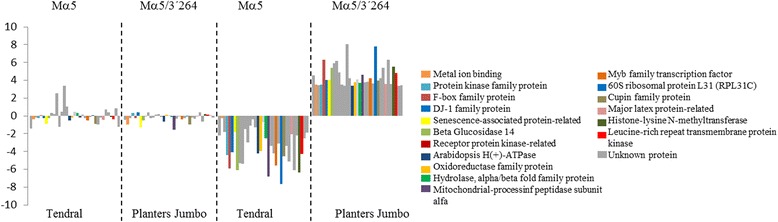
Differentially expressed genes included in the cluster 2 of Planters Jumbo with fold changes above 10. The unigenes included in this cluster were activated in Planters Jumbo cotyledons infected with MNSV-Mα5/3’264 at 5 dpi but inhibited in Tendral cotyledons infected with the same virus

The 1618 genes (812 + 806) identified in relation to MNSV-Mα5/3’264 infection of Planters Jumbo, and the 2925 (954 + 1967) identified in Tendral, denote specific differences by cultivar in response to the same virus (Fig. 3a). The functional analysis of the 1618 genes deregulated in Planters Jumbo identified the term “potassium ion transport” (GO:0006813) as over-expressed, while the genes specifically deregulated in Tendral in relation to MNSV-Mα5/3’264 infection were related to functions associated to chloroplast, photosynthesis and defense response (Additional file 3). The grouping of Planters Jumbo deregulated genes in comparison to Tendral through the use of the *k-*means algorithm split them into 12 groups with different patterns of expression. In general terms, expression tendencies were similar for both cultivars. As shown before, expression levels were greater at 5 dpi as compared to 3 dpi. The analysis of GO terms of the genes included in each group identified terms such as peroxidase activity, response to oxidative stress, chitinases and protein phosphorylation, associated to upregulated genes in both cultivars (Additional file 4). Remarkably, group 2 was composed of 274 genes that were deregulated by MNSV-Mα5/3’264, whose responses at 5 dpi were completely antagonistic, activated in Planters Jumbo and inhibited in Tendral (Fig. 7). Within this group, numerous Myb family transcription factors, ethylene response elements, many “mlo” genes and auxin response genes were found (Additional file 4). As in the previous section, genes that had expression levels above 10 times as compared to their control (FC ≥ 10) were selected. In this case, cCL555Contig1, annotated as protein L31 of the 60S subunit of the ribosome, stood out, being among the most activated in Planters Jumbo but one of the most inhibited in Tendral (Fig. 8). Among these results, kinase proteins, α-glucosidases, Myb family transcription factors, F-box family proteins or Leucine-rich repeat proteins were also found (Fig. 8; Additional file 4).

#### Comparison of changes induced by MNSV with those induced by Cucumber mosaic virus (CMV) or Watermelon mosaic virus (WMV)

**Fig. 9:**
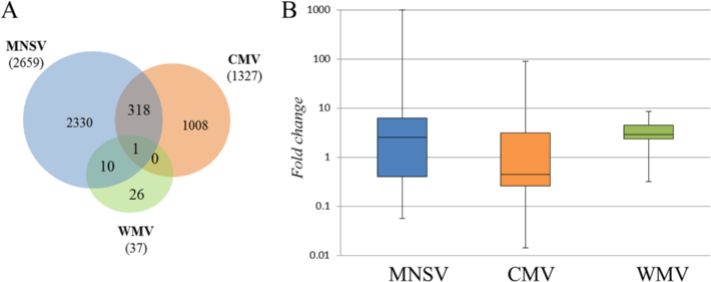
Differentially expressed genes in Tendral by three different viruses. **a** Venn diagram of the differentially-expressed genes in Tendral cotyledons infected with MNSV (blue), CMV (orange) and WMV (green) at 3 days post-inoculation (dpi) identified by SAM. Only one gene (cCL4764Contig1) was deregulated by all three viruses. **b** Broad gene expression trends. Gene expression fold changes of deregulated unigenes identified by microarray analysis were used to construct box plots for each virus

This analysis was performed with the objective of identifying common and specific transcriptomic alterations due to infection of viruses of different genera in the same host. The files corresponding to Tendral cotyledons infected by cucumber mosaic virus (CMV) and watermelon mosaic virus (WMV) at 3 dpi were extracted from publicly-available data [11, 12]. To homogenize conditions, we selected the MNSV data that corresponded to infection with MNSV-Mα5 at 3 dpi in Tendral. The data were normalized separately and were then analyzed using the SAM algorithm (Significance Analysis of Microarrays) [27]. We identified 2659 deregulated genes for MNSV-Mα5, 1327 for CMV and only 37 for WMV. Pairwise comparison identified 10 genes shared by WMV and MNSV-Mα5, which were largely peroxidases, and 318 between MNSV-Mα5 and CMV (Fig. 9a; Additional file 5). The deregulation amplitude was maximal for MNSV-Mα5, for which an apparent tendency of upregulation of genes could also be detected, while for CMV the inhibition of expression prevailed (Fig. 9b). In the case of WMV, the range of gene deregulation was much smaller than for the other two viruses, with deregulated genes showing fold changes that were positive for the most part (Fig. 9b).

Functional analysis of the genes shared by MNSV-Mα5 and CMV did not identify categories that were statistically significant. Nevertheless, manual exploration of the 318 shared genes identified transcripts that were annotated as WRKY transcription factors, peroxidases, β-1, 3 glucanases, lipoxigenases, histones or heat-shock proteins, among others (Additional file 5). A comparison of functional categories deregulated by each virus showed important differences among them. MNSV-Mα5 deregulated a great number of GO categories, with emphasis on processes linked to peroxidation, carbohydrate metabolism and responses to various types of stress. However, on the list of CMV-deregulated genes, only two GO categories were found, among them, “sequence-specific DNA binding transcription factor activity” (GO:0003700), which includes a great number of transcription factors related to hormonal response regulation (Additional file 5). Among these transcription factors, a few of the Basic Leucine Zipper Domain (bZIP) type, involved in the mediation of the response to salicylic acid, were activated, while many WRKY and Ethylene response factors (ERF) were repressed (Additional file 5), evidencing the early regulation on the hormonal responses that CMV exerts. Among the genes regulated by WMV, we identified processes related to oxidative stress as being over-represented (Additional file 5).

**Fig. 10:**
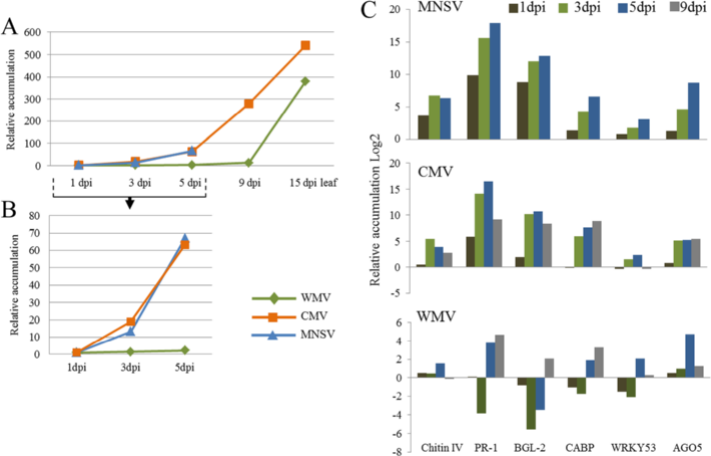
Comparison of the dynamics of RNA accumulation of MNSV-Mα5, CMV and WMV in Tendral cotyledons at 3 dpi. **a** Relative quantification of viral RNA accumulation as a function of time from MNSV, CMV and WMV-infected melon cotyledons. Infected samples at 1 day post inoculation (dpi) were used as calibrators for relative quantification for each virus. Sampling of cotyledons was done until 5 dpi for MNSV and 9 dpi for CMV and WMV. Sampling of second systemic infected leaf was done at 15 dpi for CMV and WMV-infected plants. **b** RNA accumulation in samples at early stages (1 to 5 dpi) is shown in the insert at a different scale. **c** RT-qPCR quantification of the accumulation of different genes in MNSV (quantified from 1 to 5 dpi), CMV and WMV (quantified from 1 to 9 dpi) infected plants. Relative accumulation of each gene was calculated in relation to their accumulation in healthy plants for each day of sampling. The values have been log2 transformed

The changes in common to all three viruses were minimal, only sharing the deregulation of a single gene among all three viruses (cCL4764Contig1, unknown function). As the accumulation dynamics of WMV [12] was predictably different from CMV and MNSV, we hypothesized that the dynamics of virus accumulation may have had a fundamental role in the transcriptomic alterations of the host plant. To test this hypothesis, the accumulation of the three viruses was measured in cotyledons of melon plants at different time points post-inoculation. Additionally, various genes that were allegedly deregulated by viral infection [28] were selected and the accumulation of their transcripts quantified at the same post-inoculation time points (Fig. 10). Viral accumulation dynamics of CMV and MNSV were very similar, accumulating to high levels within the first five days of infection (Fig. 10a and inset shown in 10b). On the other hand, WMV did not start to accumulate to important levels until 9 dpi, in agreement with previous observations [12]. The deregulation in cotyledons of the different genes showed responses that paralleled viral accumulation in the case of MNSV and CMV (Fig. 10c). In the case of WMV, the response was heterogeneous, with inhibitions observed in most of the genes at earlier times, and activations when the accumulation of RNA was more pronounced (Fig. 10c).

### Comparison of transcriptomic changes induced by MNSV in directly-inoculated leaves *vs* cotyledons

**Fig. 11:**
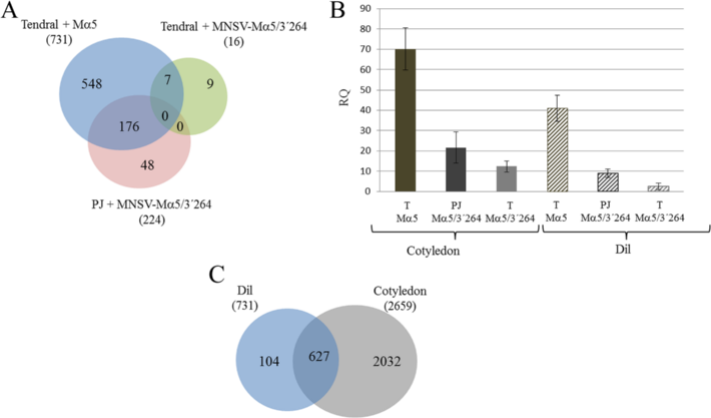
Differentially expressed genes in directly inoculated leaf (dil) of Tendral and viral load. **a** Venn diagram of differentially expressed genes identified by SAM in Tendral leaf that was directly inoculated with MNSV-Mα5 (Mα5, blue), MNSV-Mα5/3’264 (Mα5/3’264, green) and leaf of Planters Jumbo (PJ) inoculated with MNSV-Mα5/3’264 (Mα5/3’264, pink). **b** Viral load quantification in Tendral leaf inoculated with MNSV-Mα5 and MNSV-Mα5/3’264 and Planters Jumbo leaf inoculated with MNSV-Mα5/3’264 compared to the viral loading in cotyledons. Tendral cotyledons infected with MNSV-Mα5/3’264 at 1 day post-inoculation (dpi) were used to calibrate the relative quantification. **c** Differentially expressed genes by MNSV-Mα5 in directly-inoculated leaf (dil) and in cotyledons of Tendral

**Fig. 12:**
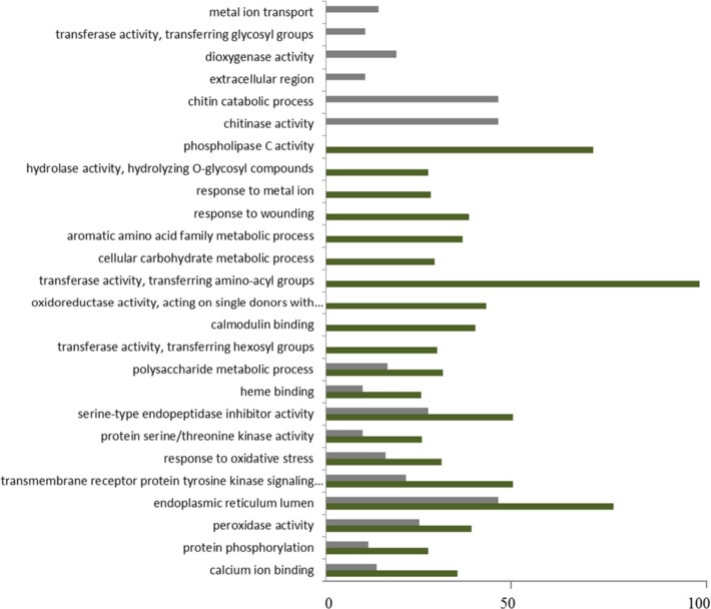
Significant Gene Ontology (GO) categories of the differentially expressed genes in cotyledons and leaves. Significant GO terms identified among the differentially-expressed unigenes identified by the microarray analysis of Tendral cotyledons at 3 dpi inoculated with MNSV-Mα5 (green) and directly-inoculated leaf with MNSV-Mα5 (grey). The percentage of deregulated unigenes from the total number of unigenes included in each GO category is indicated on the x-axis

Transcriptomic changes induced by MNSV in directly-inoculated leaves were analyzed and compared to changes in cotyledons. Sampling of the directly-infected leaves was carried out when the lesions were visible, in this case at 5 dpi. For the identification of the differentially-expressed genes, the SAM algorithm was used [27]. In Tendral inoculated with MNSV-Mα5, 731 deregulated genes were identified, while MNSV-Mα5/3’264 only deregulated 16 genes in Tendral and 224 genes in Planters Jumbo (Fig. 11a). The magnitude of the genetic deregulation in each tissue was correlated with the accumulation of MNSV as quantified by RT-qPCR, according to which the accumulation of the viral RNA in leaves was much lower than in cotyledons even at 3 dpi (Fig. 11b). A functional analysis of the deregulated genes in leaves identified many statistically significant GO categories that were mostly represented in cotyledons as well (Additional file 6). The direct comparison with the genes deregulated by MNSV-Mα5 in cotyledons at 3 dpi showed that more than 85 % of the genes were shared by both tissues (Fig. 11c). Likewise, the functional analysis of both lists of MNSV-Mα5-deregulated genes in both tissues identified various shared terms. Among the over-represented GO terms linked to cellular components, the endoplasmic reticulum lumen was important (Fig. 12). Altogether, MNSV induced a transcriptomic response in leaves that was of lesser magnitude as compared to that in cotyledons, or probably of slower progression, but which essentially involved the deregulation of the same processes in both tissues.

## Discussion

In this work, we have compared transcriptomic profiles of melon plants from two different cultivars infected with two different strains of MNSV. Melon cultivars differed on their alleles at the *nsv* locus, which encodes the eIF4E gene that controls susceptibility to most MNSV strains. The viral strains differed on their 3′-UTRs, which have been shown to control translational efficiency of MNSV RNAs and, hence, resistance-breaking capabilities.

### Comparison of profiles associated with two strains of MNSV that differed in their 3-UTRs

Our results showed that accumulation of MNSV-Mα5/3’264 was lower to that of MNSV-Mα5 in Tendral tissues, in agreement with previous observations [19, 20, 23]. As the accumulation levels of viral RNAs have been related to the intensity of the transcriptomic changes induced by certain viruses [29, 30], the reduced transcriptomic impact induced by MNSV-Mα5/3’264 at 3 dpi (Fig. 3b) could be thus explained. This is an important aspect, because other observations with both strains at discrete time points could be, at least partially, due to differences in their infection dynamics. For this reason we decided to do our study at two different time points and compare the expression patterns as a function of time for both strains.

Both viruses activated genes involved in defense responses, oxidative stress and secondary metabolism or ubiquitin-dependent catabolic processes. Primary metabolism processes, mainly photosynthesis and genes related to the chloroplasts and the organization of the cell wall, were over-represented among the genes inhibited by both viruses, leading to changes that have consistently been described for other viruses [5, 31–33]. However, there were striking differences that were mainly found in two groups of genes that had a level of activation or inhibition in response to MNSV-Mα5/3’264 that were well above the levels of those induced by MNSV-Mα5 infection. Among these genes, various transcripts of unknown unigenes (sSSH1G12_c) were found, as well as transcripts that coded for resistance-related proteins (cCL2022Contig2), proteins involved in lipid transfer (cCL5847Contig1), cytochrome p450 CYP87A3 (cCL2810Contig1), or membrane steroid binding proteins (c46d_14-E05-M13R_c), all belonging to a diversity of functional classes. These differences suggested differential interactions of both viral strains with the corresponding factors of the host, or at least with the routes and/or processes where these factors were involved. It is important to note that the region that was exchanged between the two viral strains does not code for any protein, but contains RNA structural elements that have critical regulatory functions in a variety of viral processes, including translation, replication and transcription of sub-genomic RNAs [19–21, 23, 34]. The potential functions of the altered genes for either the virus or the host are unknown, as are the regulatory mechanisms that control the expression of these genes. In this regard, our attempts to identify common sequences shared by transcripts and viral 3′-UTRs that may point toward regulation through small RNAs have not been successful.

### Comparison of profiles associated with different varieties of melon

In agreement to previous data [20, 23], MNSV-Mα5/3’264 reached higher levels of accumulation in Planters Jumbo than in Tendral. However, the amplitude of the transcriptomic response of Tendral was greater than in Planters Jumbo. The genes deregulated only in Tendral were mostly related with defense processes and functions involved in photosynthesis in general and photosystem II specifically. The decrease in photosynthetic activity has been related to defense mechanisms through the production of reactive oxygen species (ROS) derived from the chloroplasts [35, 36], and this has also been related to inhibition of photosystem II proteins with the increase in concentration of specific viruses, suggesting their involvement in basal defense responses [37]. In this sense, the inhibition of these processes in Tendral and in Planters Jumbo could be related to the launching of different basal defense responses by the different cultivars. The list of genes that were specifically deregulated in Planters Jumbo, however, was enriched in functions related to potassium ion transport, which could be involved in early signaling of events that take place during viral infection in this cultivar [38].

Planters Jumbo is a cultivar that is susceptible to MNSV-264 and MNSV-Mα5/3’264 but resistant to other strains [23, 39], with the resistance being due to a mutation in *eIF4E* [21]. The two cultivars tested here differ in this resistance gene, but presumably also in several other genes, as they are not isogenic lines. Thus, perhaps not surprisingly, both cultivars responded differently to infection by MNSV-Mα5/3’264. In this regard, the most remarkable finding was the unequivocal identification of a set of genes that showed antagonistic expression tendencies between both cultivars. Notably, clues of cultivar-viral strain double interactions were found: Among the deregulated genes, many of them coincided with those inhibited in Tendral that did not suffer changes in plants infected with MNSV-Mα5, which suggested the importance of those genes in the infection processes by MNSV-Mα5/3’264 and its differential behavior depending on the melon genotype. Among the different genes included on the list, we found Myb factors (cCL4076Contig1), which are involved, together with WRKY transcription factors, in the modulation of the plant’s hormonal expression. These factors are frequently modified as a consequence of viral infections in relation to the alteration of hormonal expression of the plant in favor of the virus or as an integrated part of the plant’s defense system [40]. Other annotated genes included diverse protein kinases, which are an essential part of the signaling events required during defense responses, as well as related to cellular death associated to resistance [41–43]. The unigene cCL555Contig1, annotated as coding for the L31 protein of the 60S ribosome subunit, showed the greatest activation in Planters Jumbo, and was found among those that were most inhibited in Tendral, becoming a very interesting candidate for the analysis of its implication in the MNSV cycle of infection [44]. Altogether, these genes represent potential targets for functional studies during infection with MNSV-Mα5/3’264 and point to the differential involvement and regulation of metabolic processes between both cultivars. Further research could include transcriptomic profiling of melon isogenic lines that only differ on *eIF4E* after MNSV-Mα5/3’264 infection.

### Comparison of profiles associated with different melon tissues

A possible criticism of the generic analysis of transcriptomic profiles could come from the assumption that the different cells or tissues respond in similar ways to viral infections, without taking into account specific alterations in the tissue as well as space-time variations of lesser intensity [30, 45]. In this sense, the transcriptomic deregulation induced by MNSV in leaves was lesser than that in cotyledons. This was probably related to the progression of infection, which was slower in leaves than in cotyledons as shown by the levels of viral accumulation. However, there was a big overlap in the nature of the genes deregulated in leaves with those from the cotyledons. Likewise, the biological functions and metabolic processes among the deregulated genes in infected leaves mostly identified the same over-represented functions in both of these tissues. These results provide validity to the general view of MNSV infections obtained in melon cotyledons.

### Comparison of profiles associated to infection by three different viruses

This comparison was possible due to the existence of previous data from research on infections of Tendral melon plants by CMV and WMV [11, 12]. As each data set come from independent experiments and with the objective of minimizing variations that could be attributed to each process of analysis, the data sets were normalized and analyzed separately following the same criteria for all three cases. After the analysis, a single gene was identified, annotated as *cytokinin-O-glucosyltransferase2*, which was deregulated by all three viruses, but with an expression pattern that differed according to the virus, that is, activated by MNSV and WMV but inhibited by CMV. Although the exact roles that cytokynins may play in plant-pathogen interactions are unknown, in Arabidopsis the involvement of these hormonal routes in some responses mediated by R proteins have been identified [46]. The common deregulation of this gene by the different viruses could indicate its involvement in the response to viruses in melon plants [47].

Among MNSV and CMV, however, numerous shared genes were identified, among them, genes related to response to stress and general defense, heat shock proteins (cCL5861Contig1), glutathione S-transferases (cA_23-D09-M13R_c), transcripts that code for resistance proteins (cCL1320Contig1), or many WRKY transcription factors, which have been identified as a response to various viruses in other hosts [5, 48]. However, each virus-host interaction was unique in terms of modified biological functions, as well as in the levels of genetic deregulation. For example, CMV caused a rapid deregulation of genes related to hormonal routes, while MNSV induced a rapid defense response and the activation of oxidative stress routes in infected plants. Unlike the other two viruses, the amplitude of the transcriptomic response induced by WMV was small, only activating oxidative-stress genes. A hypothesis that could explain the scarce genetic deregulation by WMV could be that the degree of response was related to the levels of viral accumulation. The quantification by RT-qPCR of a group of stress-response genes as a function of time showed the existence of correlation between the viral accumulation and the values of gene deregulation. These results suggest the existence of potential methodological errors when performing comparative analysis that use a single time point of observation or narrow temporal windows to compare different infections. Besides the dynamics of viral accumulation, the common deregulation of specific genes by different viruses has also been related to the phylogenetic distance of the viruses compared [49], so that new analysis and comparisons through the use of wider temporal windows and genetically-related viruses could provide more information on the processes that are commonly deregulated as a response to viral infection in melon plants.

## Conclusions

By comparing transcriptomic profiles of plants from the same cultivar infected with each of the two viral strains, we have shown that there are common but also strain-specific changes, the latter referring to a variety of genes with very different functions that were affected. No obvious regulatory features shared among deregulated genes were identified. Similarly, by comparing transcriptomic profiles of plants from each of the two cultivars infected with the same viral strain, common but also cultivar-specific changes were identified. Again, no obvious features among deregulated genes arose, but our analysis suggested the launching of different basal defense responses resulting in differential involvement of hormonal and stress response processes. An important methodological aspect emerging from this work is the influence of infection dynamics in transcriptome profiling. When comparing different viruses or viral strains, observations done at single time points could be significantly influenced by different infection dynamics. Biologically relevant data can be obtained by performing observations at different time points and comparing expression patterns as a function of time.

## Methods

### Plant material, viral isolates and virus inoculation

Melon (*Cucumis melo* L.) seeds from the cultivars Tentral (Fitó Seeds, Barcelona, Spain) and Planters Jumbo (accession C46 from the Experimental Station of “La Mayora”-CSIC, Malaga, Spain) were used. The seeds were germinated in Petri dishes for 48 h at 25 °C. After germination, the seedlings were transplanted onto 35-cell trays with soil, and grown at 27/19 °C day/night conditions. Mechanical inoculations were done on fully-expanded cotyledons after 7 days in the greenhouse or on the first fully-expanded leaves after 15 days. For these inoculations, a mix of fresh inoculum in a 30 mM potassium phosphate buffer (pH 8.0) with active charcoal and 0.037 mm Carborundum particles was used. The inocula used were from infected melon plants which had been infected with lyophilized material from MNSV-Mα5 and MNSV-Mα5/3’264 [23].

### Experimental design and sampling

The biological assay consisted of cv. Tendral plants inoculated either with MNSV-Mα5 or MNSV-Mα5/3’264, and Planters Jumbo plants inoculated with MNSV-Mα5/3’264. For the healthy controls, plants from each cultivar were treated with virus-free buffer as used for the inoculations. The sampling was done at 1, 3 and 5 days post-inoculation (dpi) in cotyledons and at 5dpi in inoculated leaves. We used three biological replicates that were composed of a pool of three different plants. Therefore, a single treatment (virus/cultivar/sampling time point) included at least 9 different plants. Viral accumulation was quantified in cotyledon samples inoculated with the different viruses (MNSV-Mα5, CMV, WMV) at 1, 3, 5 and 9 dpi. At 15 dpi, the second leaf showing systemic infection from plants inoculated with CMV-fny [50] and WMV-M116 [51] was also sampled.

### RNA extraction and microarray hybridization

All the samples were independently harvested and frozen in liquid N_2_ and stored at -80 °C. The RNA extractions were performed with Tri-Reagent (Sigma-Aldrich, St. Louis), according to the manufacturer’s instructions. After the extraction, the RNA was analyzed by dot-blot to check for the presence of the virus in the infected samples. To eliminate traces of genomic DNA, total RNA was incubated with DNAse I (New England Biolabs, London) for 10 min at 37 °C. The reaction volume was adjusted to 100 μl, and the aqueous phase was extracted with phenol/chloroform/isoamyl alcohol (25:24:1). Lastly, the RNA was precipitated with 10 % (v/v) NaOAc (3 M) and 2.5 volumes of absolute alcohol by centrifugation (12,000 x *g*, 20 min at 4 °C). The quality and quantity of RNA was verified with a ND-1000 spectrometer (Nano Drop Technologies, Wilmington, DE, USA) and a Bioanalyzer (Agilent Technologies, Palo Alto, CA, USA). For this work we used the melon microarray [11] adding 244 new unigenes [52], already used in other research studies [12, 13, 52]. In total, the melon microarray contain 17,443 melon unigenes that represent 10,649 genes in the melon genome, with additional 2,021 unigenes with no assigned hit in the annotated melon genome (Additional file 7: Table S7) [52]. Hybridizations were performed by NimbleGen’s microarray hybridization service (IRB Functional Genomics Core, BaldiriReizac, 10-12, 08028, Barcelona, Spain).

### Data analysis

The hybridization data were obtained from our experiment and from public repositories. Our experiment data provided by NimbleGen, were grouped for normalization into two groups: cotyledon and leaf. As the CMV data came from an older platform, each hybridization data group (CMV, WMV and MNSV-α5 at 3dpi) was independently normalized for later analysis and comparison. Each group of data were normalized and transformed to a log_2_ scale using the RMA (Robust Multi-array Average) algorithm found in the oligo package [53] of Bioconductor (http://www.bioconductor.org). For the cotyledon time-course experiment, the maSigPro package [24] was used to identify differentially-expresed genes. This program uses a two-regression step strategy. In the first step, a general regression model is defined. Then, the defined model is adjusted to the data through least squares, and the genes that significantly differ from this regression model are identified by correcting with a specific false discovery rate (FDR) of 1 % (Q = 0.01). In the second step, a stepwise regression is employed, and a probability (p) is calculated for each variable, showing the probability that causes the deviation. After the analysis, a list of the differentially expressed genes is obtained according to each variable (“TIME”, “TIME x Virus” and “Virus vs. Control”). We discard the differentially expressed genes associated with only the variable “TIME” in order to select those genes that were deregulated with time and associated with the virus in each cultivar. A maSigPro analysis was conducted for each cultivar. For the single stage experiment, the identification of differentially expressed genes was done through the SAM (Significant analysis of microarrays) module [27] found in the Multi Experimental Viewer (MeV, v. 4.9.0) program [54], using a FDR = 0. Genes with a fold change smaller than 2 (FC ≤2, cut-off of log_2_ ≤ 1) were filtered out.

Samples were grouped with the PCA module from MeV [54]. Clustering of the samples was done with Euclidean distance by hierachical clustering [26] and the bootstrap was done by Support trees [55] (bootstrap 100 replicates). Genes were clustered by their expression pattern by using the *k*-means clustering method [56] and Pearson’s correlation for the calculation of distances. Lastly, the functional analysis was done with the Blast2GO program [25], extracting the over- or under-represented GO terms among the differentially-expressed genes from each condition by the application of Fisher’s test (p-value <0.05).

### Microarray validation and real time quantitative reverse transcription PCR (RT-qPCR)

The melon microarray was validated in previous works [11–13] and we undertook further verification by comparing microarray and RT-qPCR expression patterns of a pathogen response protein, a calmodulin-binding protein, a lipoxigenase and a glucosyl transferase transcript. The same RNA samples from cotyledon hybridized to the microarray were used for this purpose. Data from RT-qPCR were transformed to a log_2_ scale to make the data comparable with microarray results. A strong positive correlation was found between the two sets of values (*R*^2^ = 0.86; correlation coefficient of 0.93) (Additional file 8), confirming previous results [12, 13].

For real time quantitative PCR, the first strand cDNA was synthesized using 1.5 μg of total RNA, following the directions of the reverse transcriptase manufacturer (Roche) with an oligo-dT(16) as reverse primer. As MNSV and CMV do not have a poly(A) tail, reverse primers for the respective viruses (CE-948, 5′-CCCACTATCATCACGATCTTTAC-3′, and CE-169, 5′-CCGCTTACGATTCCCAACTGT-3′) were added for transcription of the viral RNAs. The qPCR for the quantification of messenger RNA, as well as viral accumulation was performed on an AB7500 System (Applied Biosystems), using SYBR Green PCR Master Mix (Applied Biosystems) as the detector and ROX as the passive reference. All the reactions (final volume of 20 μl) contained 10 μl Master Mix, 0.15 μl of each primer (100 mM) and 60 ng of cDNA. Each reaction was done in triplicate, along with controls without DNA (NTC), using a two-step amplification protocol and adding a melting curve. The analysis of the melting curves and the NTC were done in order to ensure the specific amplification of the product and the absence of dimerization of the primers. The primers used for amplification of the target and reference genes are listed in Additional file 9.

For calculating the relative quantification of each transcript, we used the 2^ΔΔct^ method. The relative expression levels were determined through the normalization of the samples with mRNA from cyclophilin (cCL3169Contig1) as an internal control and relating it to the expression values of the healthy controls. The analysis was carried out with the SDS-7500 software and exported to a spread sheet for further calculations. The specific primer pairs were designed with Primer Express software v3.0 (Applied Biosystems). The efficiency of each primer pair was calculated through the equation: Efficiency (%) = (10^[-1/slope]^ - 1) x 100 (Guide to performing relative quantitation of gene expression using real-time quantitative PCR, Applied Biosystems).

## Additional files

Additional file 1: Table S1. Differentially expressed genes in relation to MNSV infection. Deregulated genes associated with MNSV-Mα5 and MNSV-Mα5/3’264 as a function of time in Tendral and associated with MNSV-Mα5/3’264 as a function of time in Planters Jumbo. Gene Ontology terms (GO terms) that are statistically over or under-represented on each list of differentially expressed genes are listed in contiguous spread sheets. (XLSX 245 kb) Additional file 2: Table S2. Gene Ontology terms (GO terms) of the different clusters obtained in Tendral cotyledons. Significant GO terms from the different clusters obtained according to the differentially expressed gene pattern in Tendral cotyledons infected with MNSV-Mα5 and MNSV-Mα5/3’264. % enrichment means the representation of the GO term in relation to the complete array. Genes belonging to each cluster are listed in contiguous spread sheets. A selection of genes from clusters 2 and 11 that had expression levels above 10 times as compared to their control (FC ≥ 10) is also listed. (XLSX 347 kb) Additional file 3: Table S3 Significant Gene Ontology (GO) categories among the cultivar-specific genes deregulated by MNSV-Mα5/3’264. Gene Ontology terms (GO terms) that were statistically over or under-represented among the 1618 genes that were specifically deregulated by MNSV-Mα5/3’264 in Planters Jumbo and among the 2925 genes that were specifically deregulated by the same virus in Tendral. (XLSX 12 kb) Additional file 4: Table S4. Gene Ontology terms (GO terms) of the different clusters obtained in Planters Jumbo cotyledons. Significant GO terms from the different clusters obtained according to the differentially expressed gene pattern in Planters Jumbo cotyledons infected with MNSV-Mα5/3’264 in comparison to Tendral cotyledons infected with the same virus. % enrichment means the representation of the GO term in relation to the complete array. Genes belonging to each cluster are listed in contiguous spread sheets. A selection of genes from cluster 2 that had expression levels above 10 times as compared to their control (FC ≥ 10) is also listed in contiguous spreadsheets. (XLSX 263 kb) Additional file 5: Table S5. List of differentially expressed genes by MNSV, CMV and WMV in Tendral cotyledons at 3dpi. The significant GO terms among each list of differential-expressed genes are also listed in different Excel spread sheets. (XLSX 212 kb) Additional file 6: Table S6. Differentially expressed genes in directly inoculated leaves with MNSV and their associated significant GO terms. (XLSX 65 kb) Additional file 7: Table S7. List of unigenes represented in the microarray. The corresponding gene code from the genome sequencing [10] and the ICuGI code (http://www.icugi.org) are given. The average expression value of each gene in the microarray is shown for all virus/host/time/tissue combination. The annotation of each gene is also shown as described in each source (genome, Melogen or ICuGI). (XLSX 10703 kb) Additional file 8: Figure S1. Microarray validation. Correlation between the microarray data and the RT-qPCR results. X-axis, fold change between infected samples and mock-inoculated samples in the microarray data. Y-axis, fold change according to the RT-qPCR results, data has been log2 transformed to make them comparable with the microarray results. There is a linear correlation between the values obtained with RT-qPCR and the microarray values (*R* ^2^ = 0.8598). (TIF 27 kb) Additional file 9: Table S8. qRT-PCR primers. Primers used in the qRT-PCR amplification of genes and virus. (XLSX 11 kb)
