# Supplementary figures and images for: Transcriptomic profiling of Melon necrotic spot virus-infected melon plants revealed virus strain and plant cultivar-specific alterations
Source: BMC Genomics. 2016 Jun 7;17:429. doi: 10.1186/s12864-016-2772-5 (PMC4897865; doi:10.1186/s12864-016-2772-5)

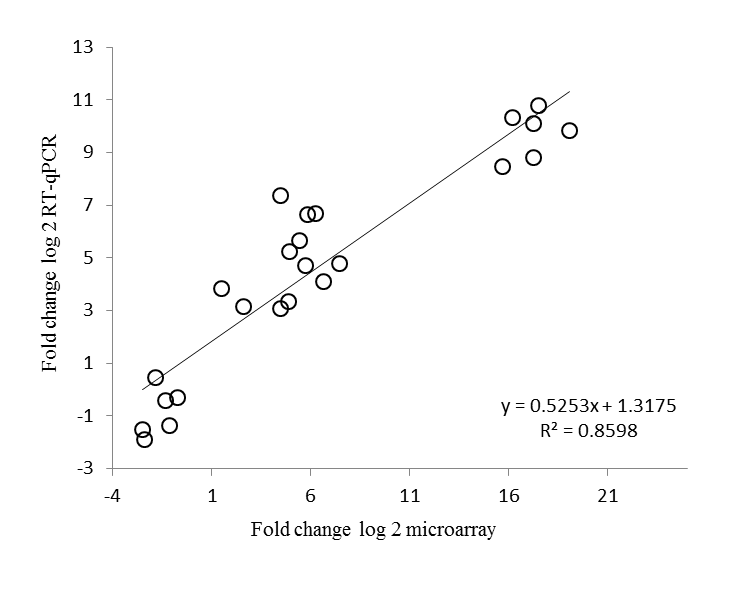

Supplement: Additional file 8: Figure S1. — Microarray validation. Correlation between the microarray data and the RT-qPCR results. X-axis, fold change between infected samples and mock-inoculated samples in the microarray data. Y-axis, fold change according to the RT-qPCR results, data has been log2 transformed to make them comparable with the microarray results. There is a linear correlation between the values obtained with RT-qPCR and the microarray values (R 2 = 0.8598). (TIF 27 kb) [file 12864_2016_2772_MOESM8_ESM.tif]
